# Cost-effectiveness of point of care smoking cessation interventions in oncology clinics

**DOI:** 10.1038/s41416-024-02819-z

**Published:** 2024-08-14

**Authors:** Kerri A. Mullen, Kelly Hurley, Shelley Hewitson, Joshua Scoville, Alyssa Grant, Kednapa Thavorn, Eshwar Kumar, Graham W. Warren

**Affiliations:** 1 University of Ottawa Heart Institute, Ottawa, ON Canada; 2 University of Ottawa, School of Epidemiology and Public Health, Ottawa, ON Canada; 3 Horizon Health Network, Fredericton, NB Canada; 4 Ottawa Hospital Research Institute, Ottawa, ON Canada; 5 New Brunswick Cancer Network, Department of Health, Fredericton, NB Canada; 6 Medical University of South Carolina, Charleston, SC USA

**Keywords:** Health care economics, Cancer, Risk factors

## Abstract

**Background:**

We examined the cost-effectiveness of providing systematic smoking cessation interventions to oncology patients at point-of-care.

**Methods:**

A decision analytic model was completed from the healthcare payer’s perspective and included all incident cancer cases involving patients who smoke in New Brunswick, Canada (*n* = 1040), cancer site stratifications, and risks of mortality, continued smoking, and cancer treatment failure over one year. Usual care (no cessation support) was compared to the standard Ottawa Model for Smoking Cessation (OMSC) intervention, and to OMSC plus unlimited cost-free stop smoking medication (OMSC + SSM), including nicotine replacement therapy, varenicline, or bupropion. Primary outcomes were incremental cost per quit (ICQ) and incremental cost per cancer treatment failure avoided (ICTFA).

**Results:**

The ICQ was $C143 and ICTFA $C1193 for standard OMSC. The ICQ was $C503 and ICTFA was $C5952 for OMSC + SSM. The number needed to treat (NNT) to produce one quit was 9 for standard OMSC and 4 for OMSC + SSM, and the NNT to avoid one first-line treatment failure was 78 for OMSC and 45 for OMSC + SSM. Both were cost-effective in 100% of 1000 simulations.

**Conclusions:**

Given the high clinical benefits and low incremental costs, systematic smoking cessation interventions should be a standard component of first-line cancer treatment.

## Introduction

Tobacco smoking remains a leading cause of premature death and places an immense economic burden on the healthcare system [1–3]. Smokers use twice as many hospital-days annually and are being hospitalized, on average, 12 years earlier than their non-smoker counterparts [4]. At least one third of all cancer deaths are attributable to smoking and smoking is causally related to over a dozen cancers [5].

Patients with cancer who smoke experience a higher risk of recurrence of primary and secondary cancers, reduced quality of life, and more cancer-related death [6, 7]. First-line chemotherapy is more effective among non-smokers, compared to current or former smokers [8]. The response to radiation treatment is poorer among patients who smoke and smokers experience more radiation-related side effects compared to former smokers and those who quit before therapy [9, 10]. Poorer surgical outcomes have been reported for oncology patients who smoke, including in-hospital mortality, pulmonary complications, surgical site infections, and hospital length of stay [11]. Quitting smoking dramatically improves health outcomes, including cancer-related events [12, 13].

A diagnosis of cancer can be life-changing, and patients with cancer who smoke report higher motivation to quit compared to smokers in the general population [11]. Unfortunately, this increase in motivation has not translated into higher quit rates due, in part, to the addictive nature of nicotine [14]. And, despite the high prevalence of smoking among patients with certain types of cancer, cessation interventions are rarely offered as part of routine cancer care.

The Ottawa Model for Smoking Cessation (OMSC) is an evidence-based, systematic smoking cessation intervention for healthcare settings that has been shown to increase smoking abstinence rates in different patient populations (e.g., cardiac, general hospital inpatient, inpatient rehabilitation, general surgery, diabetes mellitus, primary care) [4, 15–19]. Economic evaluations have found the OMSC to be cost-effective from the hospital payer perspective when delivered to patients hospitalized with cardiac or respiratory conditions [20]. Horizon Health Network (HHN), one of two regional health authorities in New Brunswick, Canada, began implementing the OMSC in 2007 in numerous healthcare settings. In 2016, implementation was expanded to oncology clinics.

Cancer-attributable healthcare costs have been increasing, due partly to the introduction of immunotherapies. Cost-effectiveness analyses are needed to help inform decisions in cancer care practice [21].

### Purpose

The purpose of this study was to evaluate the cost-effectiveness of adding cost-free stop smoking medication (SSM) at point of care for oncology patients who receive an OMSC intervention, compared to usual care (UC).

## Methods

This study was approved by the HHN Research Ethics Board (RS: 2021-2976). Reporting followed the Consolidated Health Economic Evaluation Reporting Standards (CHEERS) reporting guideline [22].

### Design, setting, and population

**Fig. 1:**
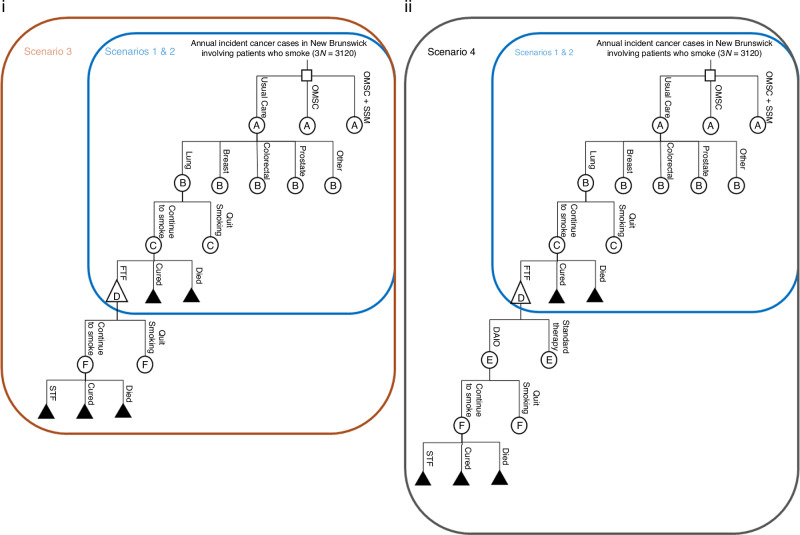
Decision analytic model. The square represents a decision node. In this case, the difference between cancer patients who smoke receiving usual care, the OMSC alone, or the OMSC + SSM is being evaluated. Circles A, B, C, D, E, and F are chance nodes and indicate where probabilities of two or more events occur. Each repeating letter within the circles (chance nodes) represents repeating events with identical probabilities. The triangles are cost end points we wish to evaluate. The solid triangles represent terminal nodes with no further action. (**i**) Figure i depicts Scenarios 1–3 from Box 1. (**ii**) Figure ii depicts Scenarios 1–2 and 4 from Box 1. Note: DAIO dual agent immunotherapy, FTF failed first-line treatment, OMSC Ottawa Model for Smoking Cessation, SSM Stop smoking medications, STF failed second-line treatment, Standard therapy chemotherapy and radiation therapy.

We conducted a cost-effectiveness analysis using a decision analytic model (Excel, Microsoft Corporation, USA). The model combined national cancer monitoring data [23], smoking cessation program administrative data from three HHN outpatient oncology clinics (Saint John Regional Hospital, Moncton Hospital, Dr. Everett Chalmers Regional Hospital), and previously published smoking prevalence and cancer treatment data [24]. The model was developed from the healthcare payer’s (the New Brunswick Department of Health) perspective and included all annual incident cancer cases involving patients who smoke in New Brunswick (*n* = 1040 in each of the UC, OMSC, and OMSC + SSM arms) [23], cancer site stratifications, mortality risk, risk of continued smoking, and risk of first-line cancer treatment failures over a one-year period (Fig. 1). It was assumed that once a patient quit smoking that they abstained for the remainder of the model timeframe. We modeled the costs of cancer treatment attributable to continued smoking using four scenarios outlined in Box 1. All study costs were expressed in 2020 Canadian dollars (CAD) using Bank of Canada inflation calculator.

Box 1 Four scenarios used to model costs of cancer treatment failures attributable to continued smoking

**Table:** 

**Assumptions**
**Scenario 1**. All patients received standard first-line cancer treatment only (i.e., surgery, chemotherapy, radiation therapy) and the cost of initial phase treatment^a^ was applied to all whether they were cured, failed treatment, or died.
**Scenario 2**. All patients received standard first-line cancer treatment only (i.e., surgery, chemotherapy, radiation therapy). Initial phase treatment costs^a^ were applied to those who survived. Terminal phase costs^a^ were applied to those who died.
**Scenario 3**. All patients received standard first-line cancer treatment (e.g., surgery, chemotherapy, radiation therapy) and either: 1) were cured, 2) failed treatment and survived, or 3) died. Initial phase cancer treatment costs^a^ were applied to those who were cured and those who failed treatment and survived during the first-line treatment phase, and terminal phase costs^a^ were applied to those who died during the first-line treatment phase.Patients who failed first-line treatment and survived went on to receive second-line treatment (e.g., surgery, chemotherapy, radiation therapy). The same smoking cessation, mortality, and risk of treatment failure rates were used to estimate second-line treatment outcomes. Initial phase treatment costs^a^ were applied to those who were cured and those who failed treatment and survived during the second-line treatment phase, and terminal phase costs^a^ were applied to those who died during the second-line treatment phase.
**Scenario 4**. All patients received standard first-line cancer treatment (e.g., surgery, chemotherapy, radiation therapy) and either: 1) were cured, 2) failed treatment and survived, or 3) died. Initial phase treatment costs^a^ were applied to those who were cured and those who failed treatment and survived during the first-line treatment phase, and terminal phase costs^a^ were applied to those who died during the first-line treatment phase.Patients who failed first-line treatment and survived went on to receive second-line treatment. The same smoking cessation, mortality, and risk of treatment failure rates were used to estimate second-line treatment outcomes. Initial phase and terminal phase treatment costs^a^ were applied to 70% of lung, breast, colorectal, and other cancers, and 100% of prostate cancer patients. Dual-agent immunotherapy (DAIO) costs^b^ were applied to 30% of lung, breast, colorectal, and other cancers, assuming 30% went on to receive DAIO as their second-line treatment.

^a^Initial and terminal phase treatment costs were taken from Iragorri et al. and de Olivera et al. and adjusted to 2020 Canadian dollars.

^b^DAIO costs were applied from Chaudhary et al. [30] and Virik and Wilson [31] and adjusted to 2020 Canadian dollars.

### Smoking cessation interventions (comparators)

#### Usual care (UC) group

Prior to 2016, HHN oncology patients who smoked did not receive any proactive, systematic smoking cessation support from clinic staff. In preparation to implement the OMSC in 2016, a consecutive series of patients from participating oncology clinics was contacted 6 months after their initial clinic visit to determine smoking abstinence rates following usual care, as part of a planned pre-implementation evaluation.

#### OMSC group

In 2016, participating HHN oncology clinics began to implement the OMSC, which included clinical protocols and tools to standardize the query and documentation of smoking status of all patients. Patients who smoked were offered: 1) brief practical advice about quitting smoking; 2) prescription for quit smoking pharmacotherapy; 3) pamphlets explaining the impacts of continued smoking on their cancer treatment; and, 4) telephone follow-up support, which included automated telephone calls delivering motivational messaging and access to cessation counselors. As with the usual care group, a consecutive subsample of patients who received the OMSC was contacted 6 months after their initial clinic visit to evaluate smoking abstinence rates following the standard OMSC intervention.

#### OMSC + free stop smoking medication offered at point of care (OMSC + SSM group)

In 2019, participating oncology clinics began offering cost-free smoking cessation medication (SSM) as part of the OMSC program. Patients could choose between nicotine replacement therapy (NRT), varenicline, or bupropion. Those who received NRT were offered an initial supply of patch plus multiple short-acting NRT (e.g., gum, inhaler, lozenge) at point of care (i.e., in clinic). Those who received varenicline or bupropion were provided a prescription and program staff coordinated payment with the patient’s pharmacy. Participants who accepted the SSM received follow-up calls from a HHN clinician 7 days prior to their quit date (if applicable), and at 3, 7, and 14 days following their initial consultation, plus automated outcome calls at days 30 and 180. Patients could receive free SSM for as long as needed. A consecutive subsample of patients was contacted 6 months after their initial clinic visit to evaluate smoking abstinence rates following receipt of the OMSC + SSM intervention.

### Model data elements

#### Cancer incidence

The number of incident cancer cases involving patients who smoke was determined by multiplying annual incident cases in the province of New Brunswick by previously published smoking prevalence data (Supplementary Table S1) [23, 24]. Approximately 4980 new cancer cases occur each year in New Brunswick; an estimated 1040 of these cases (30.2% lung, 10.3% breast, 10.5% colorectal, 8.8% prostate, 40.2% other) involve current cigarette smokers.

#### Smoking abstinence

Six-month smoking abstinence rates were determined from the before-and-after evaluations conducted by HHN’s Centre of Excellence for Clinical Smoking Cessation. Three groups of consecutive patients exposed to one of the three interventions (UC, *n* = 112; OMSC, *n* = 110; OMSC + SSM, *n* = 94) were contacted 6 months after their initial clinic visit. Demographic and smoking-related data were obtained using standardized consult forms and surveys, using validated questions and data standards [25, 26]. The outcome of 6-month smoking abstinence was self-reported by participants and determined by asking “Have you used any form of tobacco in the past 7 days”. A common standard of intention to treat analysis in cessation outcomes was used; if the patient was not reached in follow-up, it was assumed they were still smoking unless they had died [27].

Logistic regression was completed to compare smoking abstinence rates between groups. Models were adjusted for baseline characteristics thought to influence the outcome (age, sex, number of cigarettes smoked per day) and covariates that differed significantly between groups. An alpha level of 0.05 and two-tails was used for all tests of significance and interval estimates were based on 95% confidence intervals.

Covariates adjusted for in the regression analyses were: age, sex (male/female), number of cigarettes smoked per day, and smoking-related cancer (yes/no). Self-reported, intention-to-treat, 7-day point prevalence smoking abstinence rates measured at 6 months were 5.2% for UC (reference group), 16.5% for standard OMSC (adjusted odds ratio [aOR] = 4.8, 95% confidence interval [CI] 1.56–14.56; *p* = 0.006) and 24.1% for OMSC + SSM (aOR = 11.2, 95% CI 3.16–39.4; *p* < 0.001).

#### Cancer treatment failures and mortality

The number of first-line cancer treatment failures (FTF) and second-line treatment failures (STF) and costs of subsequent cancer treatments (due to failure) were calculated for each group by cancer site using methods and data similar to those used by Iragorri et al. [24]. Treatment failure rates used to calculate both FTFs and STFs were 52% for patients who continued to smoke and 40% for those who quit smoking [24]. The one-year mortality rates by cancer site provided by HHN Decision’s Support and the New Brunswick Vital Statistics Database were 26.4%, 5.1%, 14.3%, 4.5%, and 12.4% for lung, breast, colorectal, prostate, and other cancers, respectively. The same treatment failure, cessation, and mortality rates were assumed for first-line and second-line treatment phases.

$$\begin{array}{cc}{{{\rm{FTF}}}}= & \begin{array}{c}{{{\rm{baseline}}}}\; \#\; {{{\rm{of}}}}\; {{{\rm{patients}}}}\; {{{\rm{who}}}}\; {{{\rm{smoke}}}}\times {{{\rm{quit}}}}\; {{{\rm{smoking}}}}\; {{{\rm{rate}}}}\times 0.40\\ +\\ {{{\rm{baseline}}}}\; \#\; {{{\rm{of}}}}\; {{{\rm{patients}}}}\; {{{\rm{who}}}}\; {{{\rm{smoke}}}}\times {{{\rm{continued}}}}\; {{{\rm{smoking}}}}\; {{{\rm{rate}}}}\times 0.52\end{array}\end{array}$$

$$\begin{array}{cc}{{{\rm{STF}}}}= & \begin{array}{c}\left(\right.[\#\; {{{\rm{of}}}}\; {{{\rm{patients}}}}\; {{{\rm{with}}}}\; {{{\rm{FTF}}}}{{\mbox{-}}}\#\; {{{\rm{of}}}}\; {{{\rm{patients}}}}\; {{{\rm{with}}}}\; {{{\rm{a}}}}\; {{{\rm{FTF}}}}\; {{{\rm{who}}}}\; {{{\rm{died}}}}]\\ \left. \times {{{\rm{quit}}}}\; {{{\rm{smoking}}}}\; {{{\rm{rate}}}}\times 0.40\right)\\ + \\ \left(\right.[\#\; {{{\rm{of}}}}\; {{{\rm{patients}}}}\; {{{\rm{with}}}}\; {{{\rm{FTF}}}}{{\mbox{-}}}\#\; {{{\rm{of}}}}\; {{{\rm{patients}}}}\; {{{\rm{with}}}}\; {{{\rm{a}}}}\; {{{\rm{FTF}}}}\; {{{\rm{who}}}}\; {{{\rm{died}}}}]\\ \left. \times {{{\rm{continued}}}}\; {{{\rm{smoking}}}}\; {{{\rm{rate}}}}\times 0.52\right)\end{array}\end{array}$$

The number of treatment failures that were attributable to continued smoking (attributable failures [AFs]) were calculated for each group by cancer site as follows:

$$\begin{array}{cc}{{{\rm{AFs}}}}= & \begin{array}{c}({\#}{{{\rm{of}}}}\; {{{\rm{patients}}}}\; {{{\rm{who}}}}\; {{{\rm{continued}}}}\; {{{\rm{to}}}}\; {{{\rm{smoke}}}}\times 0.52)\\ \mbox{-}\\ ({\#}{{{\rm{of}}}}\; {{{\rm{patients}}}}\; {{{\rm{who}}}}\; {{{\rm{continued}}}}\; {{{\rm{to}}}}\; {{{\rm{smoke}}}}\times 0.40)\end{array}\end{array}$$

The total number of FTF and STF by cancer site and by group are presented in Supplementary Table S2 and the number of AFs are in Supplementary Table S3.

#### Intervention costs

UC participants did not receive any systematic smoking cessation support; therefore, cost was assumed to be $0. Costs of OMSC and OMSC + SSM did not include expenses related to implementing or evaluating the programs, but included costs incurred by the health authority to deliver the interventions to patients. Personnel costs associated with OMSC delivery were estimated by timing each component of the intervention (e.g., time to complete in clinic consultation, mean time of follow-up calls). Registered nurses (RN) provided the in-clinic smoking cessation intervention and registered respiratory therapists (RRT) provided the follow-up calls. Hourly rates of $39 and $33, plus 20% benefits, were used for the RN and RRT wages, respectively, based on the median wages in New Brunswick in 2020 [28, 29]. Patient follow-up costs were based on the actual proportion of patients in each group who agreed to and received follow-up support multiplied by the mean time of calls multiplied by the hourly personnel rate. Actual medication costs were gathered for each participant in the OMSC + SSM group and a mean per patient medication cost was calculated. Table 1 displays the per patient and total intervention costs for each group.

**Table 1:** Costs to deliver interventions.

Intervention component	UC	OMSC	OMSC + SSM
Personnel costs to identify smoking status	$0	$0.36	$0.36
Completion of standardized smoking cessation consult form	$0	$9.85	$13.75
Data entry	$0	$2.43	$2.43
Program database/automated call fee	$0	$2.07	$9.75
Preparing SSM (initial amount and organizing subsequent doses)	$0	$0	$4.67
SSM cost	$0	$0	$81.20
Clinical follow-up support call (days 3, 7, and 14)	$0	$0	$16.85
Follow-up support and outcome call (days 30 and 180)	$0	$0.57	$2.68
Total per patient cost	$0	$15.28	$131.69
Total cost to offer to all 1040 patients who smoke	$0	$15,886	$136,931

*OMSC* Ottawa Model for Smoking Cessation, *SSM* Stop smoking medication, *UC* Usual care.

#### Costs of first- and second-line cancer treatments

The estimated costs of cancer treatments are in Table 2. Initial and terminal phase costs are estimated costs of “standard” cancer treatments (e.g., surgery, chemotherapy, radiation) based on the study by Iragorri et al. [24]. Several dual agent immunotherapies (DAIO) are approved for use in Canada and were applied as second-line treatment to a proportion of lung, breast, colorectal and other cancers in scenario 4 of our model (Box 1). DAIO costs were based on published cost analyses in lung and colorectal cancers [30, 31].

**Table 2:** Estimated per patient treatment costs ($CAD, 2020) by cancer site.

	Treatment costs		
Cancer site	Initial phase	Terminal phase	DAIO
Lung	$25,380	$62,049	$151,084
Breast	$13,971	$48,051	$135,888
Colorectal	$29,634	$57,832	$135,888
Prostate	$8423	$46,849	n/a
Other	$43,880	$65,334	$135,888

Sources: Iragorri et al. [14], Chaudhary et al. [30], and Virik and Wilson [31].

Initial phase and terminal phase costs are estimated for standard surgery, chemotherapy, and radiation costs.

*DAIO* Dual agent immunotherapies.

### Outcomes

#### Intermediate outcomes and incremental cost-effectiveness ratios

Our primary outcomes were incremental cost per quit and incremental cost per cancer treatment failure avoided. Additional outcomes included: number of first-line cancer treatments (FTF) avoided; number needed to treat (NNT) to produce one quit; NNT to avoid one FTF; number needed to quit (NNQ) to avoid one FTF; NNT to break even on the investment; and, NNQ to break even on the investment. Return on investment (ROI) was calculated. Supplementary Table S4 displays the formulas for all outcomes.

#### Sensitivity analyses

A series of sensitivity analyses were conducted to assess the robustness of our results. One-way sensitivity analysis was performed for smoking prevalence rates, mortality rates, quit rates, and program costs for all three groups. Probabilistic sensitivity analysis (PSA) was also conducted for all parameters and all groups in the model using a Monte Carlo simulation technique with 1000 iterations. Beta distributions were used for smoking prevalence rates, mortality rates, and quit rates, and the gamma distribution was assigned to cost data. The PSA results were used to create cost-effectiveness acceptability curves, which show the probability of OMSC and OMSC + SSM programs being cost-effective over a range of willingness-to-pay thresholds. A scenario analysis was conducted to explore variation in our results that resulted from the use of the number of quitters as a secondary outcome.

#### Approach to engagement with patients and others affected by the study

Semi-structured interviews were completed with a sample of patients (smoke-free at 6 months, *n* = 14; still smoking at 6 months, *n* = 17) and health providers (*n* = 14) involved in the OMSC + SSM intervention to assess importance, satisfaction, facilitators, and barriers to the program. Results are summarized in the Supplemental Material.

## Results

### Summary of main results

From a healthcare payer’s perspective, the OMSC and OMSC + SSM programs were associated with high clinical benefits (i.e., lower number of treatment failures, greater number of quitters) at a low increment in healthcare costs. The costs of cancer treatment attributable to continued smoking for each of the four scenarios from Box 1 are presented in Supplementary Table S5A–D. Compared to UC, the estimated savings realized by offering the OMSC + SSM to all 1040 smokers receiving cancer treatment in NB annually ranged from $665,227 in scenario 1 to $1,683,518 in scenario 4. This represents a ROI of between 486% and 1229%. Standard OMSC led to savings of between $386,919 and $1,055,984 relative to UC, a ROI of between 282% and 771%.

Table 3 summarizes the ICERs and additional base case outcomes.

**Table 3:** Base case results.

	OMSC	OMSC + SSM
Number of first-line cancer treatment failures avoided	13	23
Number of second-line cancer treatment failures avoided	12	22
NNT to produce one quit	9	4
NNT to avoid one treatment failure	78	45
NNQ to avoid one treatment failure	12	14
NNT to break even on the investment	15	86
NNQ to break even on the investment	2	27
Incremental cost per treatment failure avoided (reference: UC)	$1193	$5952
Incremental cost per treatment failure avoided (reference: OMSC)	–	$12,492
Incremental cost per quitter (reference: UC)	$143	$503
Incremental cost per quitter (reference: OMSC)	–	$752
Incremental cost per smoker-patient reached (reference: UC)	$15	$132

*NNQ* Number needed to quit, *NNT* Number needed to treat, *OMSC* Ottawa Model for Smoking Cessation, *SSM* Stop smoking medications, *UC* Usual care.

### Effect of uncertainty

**Fig. 2:**
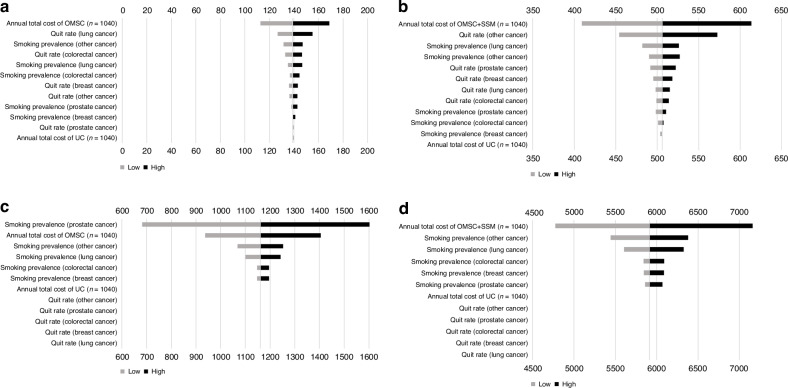
One-way sensitivity analysis: tornado plots. **a** Incremental cost per quit (OMSC vs. UC). **b** Incremental cost per quit (OMSC + SSM vs. UC). **c** Incremental cost per treatment failure avoided (OMSC vs. UC). **d** Incremental cost per treatment failure avoided (OMSC + SSM vs. UC). Note: OMSC Ottawa Model for Smoking Cessation, SSM stop smoking medication, UC usual care.

The main drivers of the incremental cost per quit findings were the annual costs of the OMSC and OMSC + SSM interventions (Fig. 2a, b). The incremental cost per FTF-avoided findings were highly sensitive to changes in smoking prevalence in the prostate cancer group followed by intervention costs, in the case of OMSC only (Fig. 2c), and highly sensitive to changes in intervention costs and smoking prevalence of the other cancer group, in the case of OMSC + SSM (Fig. 2d).

**Fig. 3:**
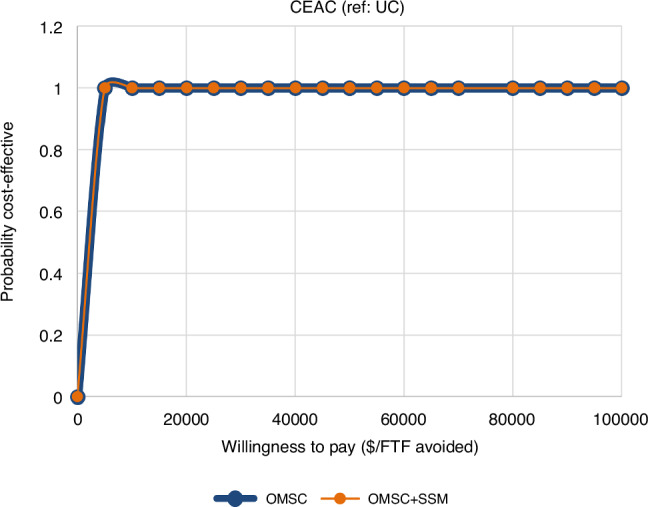
Cost-effectiveness acceptability curve (reference: usual care). Demonstrates change in the probability that the OMSC and OMSC + SSM are cost-effective as the cost per treatment failure avoided increases. Note: FTF first-line failures, OMSC Ottawa Model for Smoking Cessation, SSM Stop smoking medications.

Probabilistic sensitivity analysis indicated that OMSC and OMSC + SSM had 100% probability of cost-effectiveness compared to usual care if the health care payer was willing to pay at least $60.00 to gain at least one quit or prevent one treatment failure (Fig. 3).

Results from the scenario analysis using the number of quitters as an outcome were consistent with our base case results in that OMSC and OMSC + SSM interventions were more effective and more costly than usual care in 100% of 1000 simulations.

## Discussion

In this cost-effectiveness study, the OMSC and OMSC + SSM were associated with minimal add-on costs and greater benefits compared to no intervention when offered at point of care to oncology patients who smoke. By not offering smoking cessation support to the 1040 new cancer patients who smoke in New Brunswick each year, an estimated 106 first-line treatment and 54 second-line treatment failures would be experienced due to continued smoking, leading to $5 million - $7 million in subsequent treatment costs. In contrast, offering the OMSC + SSM would prevent 24 first-line and 23 second-line treatment failures and save >$1 million in subsequent treatment costs, compared to usual care. Per patient first-line cancer medication and radiation treatment costs in 2020 in Canada cost, on average, $5100 and $16,700, respectively [32]. Adding effective smoking cessation support that includes SSM at point of care to cancer treatment would represent less than 3% of the medication cost, and less than 1% of the overall treatment cost for cancer patients who smoke; even less when compared to the costs of DAIO (>$100,000), which are being used more every year.

### Comparison to other studies

Previous work found the OMSC to be cost-effective when delivered to cardiac and respiratory inpatients in Ontario, Canada [20], and a similar intervention, the CURE Project, to be cost-effective as implemented in hospitals in Greater Manchester, UK [33]. This is the first study examining the effectiveness of the OMSC in oncology settings. A recent study in cancer settings in the United States (US) assessed the incremental cost per quit (ICQ) of an intensive cessation intervention (11 brief telephone counseling sessions plus up to 12 weeks of free cessation medication), compared to “standard of care” cessation intervention (up to 4 counseling sessions plus medication advice) and usual care (referral to telephone quit line) [34]. The ICQ of the intensive intervention was $3906 ($5239 CAD 2020) relative to standard of care and $9866 ($13,235 CAD 2020) relative to usual care. Our ICQs were much lower at $752 and $503, respectively. Our intervention was less intensive in terms of follow-up contacts, and our mean length of SSM use was less than 12 weeks. The US study observed a similar absolute difference in 7-day point prevalence abstinence at 6 months between the intensive treatment and standard treatment groups: 34.5% vs. 21.5%, respectively (difference, 13.0% [95% CI, 3.0–23.3%]); [35] however, abstinence rates were higher than those observed in our study. Two recent simulation studies, one US and one Canadian, assessed the cost-effectiveness of offering smoking cessation as part of lung cancer screening programs, from the societal and healthcare payer perspective, respectively [34, 35]. Both found cessation interventions to be cost-effective in terms of incremental cost per QALY suggesting that cancer screening programs may be an important opportunity to intervene further upstream [36, 37].

### Limitations

We did not model changes in smoking status that can occur throughout a quit attempt, nor did we have data on the effects of the intervention on abstinence beyond 6 months. A recent effectiveness study of a similar intervention offered to head and neck cancer patients in northern Ontario, Canada found abstinence rates to be consistent at 6 and 12 months (23.7% and 23.9%, respectively), supporting our model’s assumption that abstinence rates were maintained [38].

We did not account for smoking cessation interventions that may have occurred outside the cancer care setting (e.g., in primary care) that could have generated additional healthcare costs. We chose to examine costs and outcomes specific to cancer patients and cancer treatments and did not include the effects of quitting smoking on preventing other important smoking-attributable illnesses (e.g., cardiac events, stroke, peripheral vascular disease, respiratory diseases) in our model. The inclusion of such data would likely lead to even greater cost-effectiveness. Our study only examined costs and benefits that occurred over one treatment year. More evidence is needed to determine the long-term (>1 year) impacts of smoking cessation on cancer treatment outcomes. We modeled AFs from a previous Canadian study. Having actual treatment failures would have strengthened our analysis.

### Generalizability

This study was conducted in New Brunswick, Canada – a province of approximately 790,000 residents with nearly 5000 incident cancer cases each year. Cancer care is led by the New Brunswick Cancer Network (a branch of the Department of Health) and is primarily publicly funded, whereby medically necessary services including hospital stays, diagnostic investigations, and surgical procedures, as well as chemotherapy and radiation therapy are available at no cost to residents. The costs of smoking cessation treatment, healthcare human resources, and cancer treatments were from Canadian sources and presented in Canadian dollars. The smoking cessation program reviewed was the OMSC, a systematic intervention that has been widely adopted in Canadian inpatient, outpatient, and primary care settings. While the intervention may differ from other cessation programs, the components (practical advice, pharmacotherapy, follow up) are based on contemporary clinical practice guidelines [39–42].

## Conclusion

The OMSC is both effective and associated with minimal costs when implemented in oncology settings. It is incrementally more effective when free SSM is provided. Given the effects of smoking cessation on cancer treatment outcomes, and the relatively low cost of intervention, cessation treatment should be offered as part of standard cancer treatment to patients who smoke.

## Supplementary information

Supplemental Material

## Data Availability

Study data can be made available upon request and completion of data sharing agreement.
