# Supplementary material for: Cost-effectiveness of point of care smoking cessation interventions in oncology clinics
Source: Br J Cancer. 2024 Aug 14;131(7):1178–85. doi: 10.1038/s41416-024-02819-z (PMC11443138; doi:10.1038/s41416-024-02819-z)
Supplement: Supplementary file 1 — Supplemental Material [file 41416_2024_2819_MOESM1_ESM.docx]

# Cost-effectiveness of point of care smoking cessation interventions in oncology clinics

Mullen KA, Hurley K, Hewitson S, Scoville J, Grant A, Thavorn K, Kumar E, Warren GW

**Supplementary material**

Supplementary Table S1 - Annual cancer incidence in New Brunswick Page 2

Supplementary Table S2 - Total number of treatment failures by cancer site and by group Page 3

Supplementary Table S3 - Number of treatment failures attributable to continued smoking (AFs) Page 4

Supplementary Table S4 - Intermediate and cost-effectiveness outcomes with formulae Page 5

Supplementary Table S5 A-D - Costs of cancer treatment failures attributable to continued Page 6-7

smoking (AFs)

Results of structured interviews Page 8-14

Supplementary Table S1. Annual cancer incidence in New Brunswick

| Cancer site | New cases, n | Smoking prevalence | New cases involving smokers, n |
| --- | --- | --- | --- |
| Lung | 805 | 39% | 314 |
| Breast | 595 | 18% | 107 |
| Colorectal | 605 | 18% | 109 |
| Prostate | 575 | 16% | 92 |
| Other | 2400 | 17% | 418 |
| Total | 4980 |  | 1040 |

Sources:

1. Statistics Canada. 2021. https://www150.statcan.gc.ca/t1/tbl1/en/tv.action?pid=1310011101
2. Iragorri, et al. Curr Oncol. 2020 December:27(6)307–312

Supplementary Table S2. Total number of treatment failures by cancer site and by group

|  | Usual Care | | | OMSC | | | OMSC+SSM | | |
| --- | --- | --- | --- | --- | --- | --- | --- | --- | --- |
|  | FTF | STF | Total | FTF | STF | Total | FTF | STF | Total |
| Lung | 161 | 83 | 244 | 157 | 79 | 236 | 154 | 76 | 230 |
| Breast | 55 | 28 | 83 | 54 | 27 | 81 | 53 | 26 | 79 |
| Colorectal | 56 | 29 | 85 | 55 | 27 | 82 | 54 | 26 | 80 |
| Prostate | 47 | 24 | 71 | 46 | 24 | 70 | 45 | 23 | 68 |
| Other | 215 | 110 | 325 | 209 | 105 | 314 | 205 | 101 | 306 |
| Total | 534 | 274 | 808 | 521 | 262 | 783 | 511 | 252 | 763 |

FTF = first-line treatment failure; STF = second-line treatment failure

Supplementary Table S3. Number of treatment failures attributable to continued smoking (AFs), by cancer site and group

|  | Usual Care | | OMSC | | OMSC+SSM | |
| --- | --- | --- | --- | --- | --- | --- |
| Cancer site | First-line  AFs | Second-line AFs | First-line  AFs | Second-line AFs | First-line  AFs | Second-line AFs |
| Lung | 31 | 16 | 27 | 14 | 25 | 12 |
| Breast | 12 | 6 | 10 | 5 | 9 | 5 |
| Colorectal | 11 | 5 | 10 | 5 | 9 | 5 |
| Prostate | 10 | 5 | 9 | 5 | 8 | 5 |
| All Other | 42 | 21 | 37 | 18 | 33 | 16 |
| Total | 106 | 53 | 93 | 47 | 84 | 43 |

Supplementary Table S4. Intermediate and cost-effectiveness outcomes with formulae

| **Outcome** | **Formula** | |  |
| --- | --- | --- | --- |
|  |  | |  |
| First-line cancer treatment failures avoided = | Number UC failures **–** Number of intervention failures | |  |
|  |  | |  |
| NNT to avoid one first-line treatment failure = | Total number of smokers treated | |  |
|  | Number of first-line treatment failures avoided | |  |
|  |  | |  |
| NNQ to avoid one first-line treatment failure = | Total number of quitters | |  |
|  | Number of first-line treatment failures avoided | |  |
|  |  | |  |
| NNT to break even on the investment = | Number of patients treated **×** total intervention cost | |  |
|  | Total savings due to second line treatment costs avoided | |  |
|  |  | |  |
| NNQ to break even on the investment = | Number of quitters **×** total intervention cost | |  |
|  | Total savings due to second line treatment costs avoided | |  |
|  |  | |  |
| Incremental cost per quitter = | (Total cost of intervention – total cost of UC) | |  |
|  | (# quitters with intervention – # quitters with UC) | |  |
|  |  | |  |
| Incremental cost per treatment failure avoided = | (Total cost of intervention – total cost of UC) | |  |
|  | (# failures avoided with intervention – # failures avoided UC) | |  |
|  |  | |  |
| Return on investment = | Total savings due to second line treatment costs avoided | **×** 100% | |
|  | Total cost of intervention |  |  |
|  |  |  |  |

Supplementary Table S5. Costs of cancer treatment failures attributable to continued smoking (AFs), by group

A. Scenario 1 – first-line treatments only; initial phase costs

| **Cancer site** | **Usual Care** | OMSC | OMSC+SSM |
| --- | --- | --- | --- |
| Lung | $786,780 | $685,260 | $634,500 |
| Breast | $167,652 | $139,710 | $125,739 |
| Colorectal | $325,974 | $296,340 | $266,706 |
| Prostate | $84,230 | $75,807 | $67,384 |
| All Other | $1,842,960 | $1,623,560 | $1,448,040 |
| **Total** | **$3,207,596** | **$2,820,677** | **$2,542,369** |

OMSC+SSM savings over Usual Care: $665,227; OMSC over Usual Care: $386,919

B. Scenario 2 – first-line treatment only; initial and terminal phase costs

| **Cancer site** | **Usual Care** | OMSC | OMSC+SSM |
| --- | --- | --- | --- |
| Lung | $1,080,132 | $946,637 | $891,183 |
| Breast | $201,732 | $173,790 | $125,739 |
| Colorectal | $412,004 | $324,538 | $294,904 |
| Prostate | $84,230 | $75,807 | $67,384 |
| All Other | $1,906,350 | $1,730,830 | $1,533,856 |
| **Total** | **$3,684,448** | **$3,251,602** | **$2,913,066** |

OMSC+SSM savings over Usual Care: $771,382; OMSC over Usual Care: $432,846

C. Scenario 3 – first- and second-line treatments; initial and terminal phase costs

| **Cancer site** | **Usual Care** | | | OMSC | | | OMSC+SSM | | |
| --- | --- | --- | --- | --- | --- | --- | --- | --- | --- |
|  | First-line | Second-line | Total | First-line | Second-line | Total | First-line | Second-line | Total |
| Lung | $1,080,132 | $552,756 | $1,632,888 | $946,637 | $475,950 | $1,422,587 | $891,183 | $414,567 | $1,305,750 |
| Breast | $201,732 | $83,826 | $285,558 | $173,790 | $82,088 | $255,878 | $125,739 | $55,884 | $181,623 |
| Colorectal | $412,004 | $206,002 | $618,006 | $324,538 | $170,245 | $494,783 | $294,904 | $176,368 | $471,272 |
| Prostate | $84,230 | $42,115 | $126,345 | $75,807 | $53,147 | $128,954 | $67,384 | $42,115 | $109,499 |
| All Other | $1,906,350 | $1,029,722 | $2,936,072 | $1,730,830 | $854,124 | $2,584,954 | $1,533,856 | $744,988 | $2,278,844 |
| **Total** | **$3,684,448** | **$1,914,421** | **$5,598,869** | **$3,251,602** | **$1,635,554** | **$4,887,156** | **$2,913,066** | **$1,433,922** | **$4,346,988** |

OMSC+SSM savings over Usual Care: $1,251,881; OMSC over Usual Care: $711,713

D. Scenario 4 – first- and second-line treatments; DAIO costs applied to a proportion of second-line treatments

| **Cancer site** | **Usual Care** | | | OMSC | | | OMSC+SSM | | |
| --- | --- | --- | --- | --- | --- | --- | --- | --- | --- |
|  | **First-line** | Second-line | Total | **First-line** | Second-line | Total | **First-line** | Second-line | Total |
| Lung | $1,080,132 | $1,144,607 | $2,224,739 | $946,637 | $968,143 | $1,914,780 | $891,183 | $880,714 | $1,771,897 |
| Breast | $201,732 | $327,660 | $529,392 | $173,790 | $204,578 | $378,368 | $125,739 | $177,801 | $303,540 |
| Colorectal | $412,004 | $312,256 | $724,260 | $324,538 | $266,610 | $591,148 | $294,904 | $282,622 | $577,526 |
| Prostate | $84,230 | $42,115 | $126,345 | $75,807 | $53,152 | $128,959 | $67,384 | $42,115 | $109,499 |
| All Other | $1,906,350 | $1,652,324 | $3,558,674 | $1,730,830 | $1,363,342 | $3,094,172 | $1,533,856 | $1,183,574 | $2,717,430 |
| **Total** | **$3,684,448** | **$3,478,962** | **$7,163,410** | **$3,251,602** | **$2,855,824** | **$6,107,426** | **$2,913,066** | **$2,566,826** | **$5,479,892** |

OMSC+SSM savings over Usual Care: $1,683,518; OMSC over Usual Care: $1,055,984

OMSC = Ottawa Model for Smoking Cessation; SSM = Stop smoking medication

**Semi-structured interviews**

**Summary**

Structured interviews were completed with a sample of patients (smoke-free at 6 months, n=14; still smoking at 6 months, n=17) and health providers (n=14) involved in the OMSC+SSM intervention to assess importance, satisfaction, facilitators, and barriers to the program.

Overall, 81% of patients found the telephone follow-up support helpful. When asked if they would have made a quit attempt had stop smoking medication not been offered for free by the clinic, the majority responded no (55%) or maybe (15%). Among the patients surveyed who were still smoking (n=19), 89% felt that this quit attempt would lead to another quit attempt in the future.

Among health providers surveyed (n=14), 100% felt that providing smoking cessation services is an important clinical practice and 100% believed it’s a service that is valued by oncology patients and their families. The majority (92%) felt that offering free stop smoking medication and having patient follow-up support provided by a HHN clinician increased their likelihood of offering smoking cessation services to patients. 100% felt the free medication and follow-up support increased the value and quality of the smoking cessation program. Time was the main challenge identified in providing medications at point of care within the oncology clinic.

**Patient Respondents:**

*Respondents who were smoke-free at 6-month follow-up: n=14*

*Respondents who were still smoking at 6-month follow-up: n=17*

1. Before you came to the cancer clinic, did you know that smoking during chemotherapy and radiation treatment can change how well your treatment works?

2. Was information about the impact of smoking on chemotherapy and radiation treatment shared with you by the clinic?

“I learned that my cancer treatment will be 40% more effective if I quit.”

“They talked about the importance of quitting on my treatment.”

“It helped inform my decision to quit.”

“My diagnosis was the deciding factor, but the stats solidified my decision.”

3. Did having follow-up support 7 days before your quit date, 3,7, and 14 days after your quit date helpful?

“Yes, it was nice to know someone cared.”

“The calls were motivating.”

“It was encouraging and answered a lot of my questions.”

“I appreciated the support.”

“It gave me some accountability.”

“Yes, they kept me on track.”

“The tips and tricks were helpful. I found it encouraging and was good to be held accountable.”

“Not really. I had already decided.”

“Not really. I was doing well on my own.”

“She gave information, but it didn’t help with my cravings.”

4. Would you have made a quit attempt if stop smoking medications were not provided by the cancer clinic?

“I wouldn’t have quit right then.”

“No. The fact that it was free was good.”

“The free smoking cessation medication made the decision for me.”

“I knew I had to quit, but the meds were helpful.”

“Yes, but I quit frequently.”

“Yes. The cancer was a scare, but the free meds made it that much easier.”

“Yes, I didn’t use any of the NRT.”

**For those not smoke-free at 6 month follow up (n=19)**

5. Even though you were not successful in quitting smoking, do you feel that this quit attempt may lead to another quit attempt in the future?

“Yes, if medication was free.”

“I was smoke-free until about a month ago… [described a traumatic experience]…I had no other way to cope so started smoking again. I want to quit again. I know what meds to use.”

“Yes. I’ll try again when the time is right. My family limits me to 3 cigarettes per day. I’m not really interested in cutting more than that.”

“Yes, I’d like to try again.”

“Yes. I have to quit.”

“Yes, I’ve already started Champix.”

“Yes, I’m ready to quit. My prescription starts next week.”

“Yes. But I don’t need the free NRT. I bought a vape and am going to try that.”

“Maybe. Free meds wouldn’t make a difference for me.”

“No. My age and circumstances, I probably won’t quit.”

**For those who *were* smoke-free at 6 month follow up**

6. Are there any other factors you feel contributed to your success in quitting smoking?

*Six (6) respondents reported family, friends, and society helped:*

“My family.”

“Society. No one smokes anymore.”

“Being strong. Support from my friend.”

*Five (5) respondents reported health and treatment-related factors:*

“I’ve already started to feel better, so I know it was the right decision and will keep going.”

“I had a chest infection that lasted a long time. It helped me realize, I need to quit.”

“Just my diagnosis.”

“When I was in hospital. I didn’t smoke at all after my operation. I came home, put a patch on, and haven’t smoked.”

“Cancer was my wake-up call. I’ve never been sick before.”

“No. Just knowing that smoking can impact my cancer treatment was enough.”

*Other factors:*

“I’m stubborn.”

“The inhaler.”
